# Gait Parameters of Women Before Knee Joint Arthritis—Analysis Using the MoKA System

**DOI:** 10.3390/s26010136

**Published:** 2025-12-25

**Authors:** Maciej Kuś, Dagmara Wasiuk-Zowada, Katarzyna Herman, Jerzy Cholewiński, Andrzej Knapik

**Affiliations:** 1 Doctoral School, Faculty of Health Sciences in Katowice, Medical University of Silesia, 40-752 Katowice, Poland; d201139@365.sum.edu.pl; 2 Department of Physiotherapy, Faculty of Health Sciences in Katowice, Medical University of Silesia, 40-752 Katowice, Poland; 3 Department of Rehabilitation, Faculty of Health Sciences in Katowice, Medical University of Silesia in Katowice, 40-635 Katowice, Poland; kherman@sum.edu.pl (K.H.); jcholewinski@sum.edu.pl (J.C.); 4 Department of Adapted Physical Activity and Sport, Faculty of Health Sciences in Katowice, Medical University of Silesia, 40-752 Katowice, Poland; aknapik@sum.edu.pl

**Keywords:** knee osteoarthritis, 6MTW, IMU, gait analysis

## Abstract

Knee osteoarthritis significantly reduces quality of life due to difficulties with locomotion. The objective assessment of gait parameters can provide guidance for developing therapeutic programs, and wearable sensors are becoming increasingly common for this purpose. The Movement Kinematics Analysis System (MoKA) utilizes the Inertial Measurement Unit, which enables gait analysis in non-laboratory settings. The aim of the study was to determine gait parameters in women scheduled for knee replacement immediately before surgery, along with determining the measurement reliability. Seventy-six women were enrolled in the study (research group n = 25; control group n = 51). The participants completed the 6MWT with gait monitoring via the MoKA system. A comparison of pain intensity before and after the 6MWT revealed differences in *p* < 0.001. A comparison between the groups revealed differences in distance and step count. Pain intensity was negatively correlated with distance (R = −0.44) and the number of steps (R = −0.44), but did not affect the average length of steps (R = 0.05). The overall consistency assessment (AC) demonstrated good internal consistency. The qualitative ICC assessment indicated moderate reliability for three measurements, good for one, and excellent for the remaining measurements. It can be assumed that the assessment of biomechanical gait parameters using a system equipped with an IMU meets the criteria for measurement reliability. The gait of women with KOA scheduled for total knee replacement surgery is flattened and slowed, which may provide guidance for the use of appropriate postoperative exercises to achieve appropriate gait biomechanics.

## 1. Introduction

Osteoarthritis (OA) is a common musculoskeletal disorder with a global reach, which, together with its frequency, places a significant burden on healthcare systems [1,2,3]. Age is a significant factor influencing the occurrence of degenerative changes—it is estimated that it affects 35% of the population over 65 years of age with varying degrees of symptom severity [4,5]. OA is currently believed to be caused by a complex interaction of genetic, cellular, biomechanical, and immunological factors that lead to a loss of physiological balance between the catabolic and anabolic processes occurring in joint cartilage and other joint components [6]. Some authors explicitly associate OA with a metabolic syndrome and demonstrate a similar course of the disease in terms of the presence of cytokines and factors characteristic of chronic inflammation [7,8]. Excess body weight is a predisposition to OA. It is estimated that 40% of cases occur in women with an elevated BMI, most often in those older in age [6].

The role of optimal physical activity levels and targeted exercise therapy is emphasized in the prevention and relief of OA symptoms [9,10,11]. However, intensive participation in a number of sports disciplines, e.g., American football, soccer, rugby, handball, wrestling, and ice hockey, increases the risk of OA, which predicts more frequent interventions among younger individuals [12,13]. OA develops over the long term, and despite the use of various strategies addressing modifiable risk factors, joint replacement is often the only effective solution [14].

Knee osteoarthritis (KOA) is a chronic, progressive disease that affects joints and their surrounding tissues. Initially, damage occurs in the joint cartilage, followed by the subchondral bone and synovial structures. KOA causes pain and stiffness that reduce quality of life and interfere with daily activities such as rising from a chair, climbing stairs, and walking, leading to significant disability [15,16,17]. KOA places a significant burden on healthcare systems [1,2,3]. Globally, it affects over 250 million people [18]. Age is a significant factor in the occurrence of degenerative changes, and it is estimated that 35% of the population over 65 years of age suffers from KOA, with varying degrees of symptom severity.

Currently, the most commonly used methods for assessing the severity of osteoarthritis and its effects are self-assessment questionnaires, clinical assessment of function, and—if available—gait analysis using specialized equipment [19,20,21]. Popular scales are convenient to use and combine measurements of pain and function. A critical evaluation of these scales primarily considers the predominance of pain over function. Therefore, results based on functional tests are considered more objective [22,23]. Their results allow for predicting the rate of patient recovery after surgery [20]. The use of various types of gait analysis equipment, including solutions based on treadmills with optical sensors, is associated with certain limitations, including the need to purchase expensive equipment and to engage specialists to operate it, as well as the duration of the study. Another limitation is the laboratory nature of the study, which invariably affects the results. For example, creating the optimal conditions possible, taking into account lighting quality and limiting occlusion, can pose certain challenges in obtaining appropriate data [24,25]. The limited space available in laboratory spaces can also alter the natural gait of the subjects, which would impact gait parameters.

Therefore, the use of body-worn inertial measurement systems has many advantages. Previous reports indicate that measurements using IMU (Inertial Measurement Unit) sensors are as accurate as those using optical sensors, and their advantages include the absence of the aforementioned limitations related to laboratory testing, low cost, and ease of use (transferability) [19,26], which makes IMUs currently the most commonly used sensors for gait analysis [27,28,29]. Currently, based on a growing number of empirical studies, there is an ongoing discussion on the optimal location of these sensors on the human body and overcoming technological limitations [30,31,32,33].

Integrating sensors with an appropriate system creates new research opportunities. Objective analysis of musculoskeletal dysfunction in patients with KOA, both before and after surgery, can provide valuable information regarding individual treatment strategies and the patient’s recovery [34,35].

The aim of the study was to determine gait parameters in women undergoing knee replacement due to knee degeneration immediately before surgery and to compare them with those without musculoskeletal dysfunction. The reliability of the measurements was also assessed.

## 2. Materials and Methods

### 2.1. Participants

A total of 76 women aged 49–76 years were examined (mean 72.1; median 72.5; SD = 7.9). The research group, RG (n = 25), consisted of women who qualified, and were admitted to the hospital department of trauma and orthopedic surgery for knee replacement due to advanced degenerative changes. Their age (mean 70.2; median 71.0; SD = 8.40) and BMI (mean 31.2; median 31.2; SD = 6.0) were measured. In this group, 10 people (40%) had planned surgery on the left knee, 15 people (60%) on the right. The level of pain at rest was determined immediately before the tests (VAS) (mean 3.8; median = 3.0; SD = 2.9), and pain was assessed immediately after the test (mean 5.04; median = 4.5; SD = 2.7).

The control group (CG) consisted of 51 women without musculoskeletal complaints. The recruitment for the study was conducted among participants at the University of the Third Age (age—mean 73.3; median 73.0; SD = 7.4; BMI—mean 28.0; median 28.6; SD = 4.9). A comparison of the groups revealed no differences in either age or BMI (*p* > 0.05).

### 2.2. Experimental Setup

**Figure 1:**
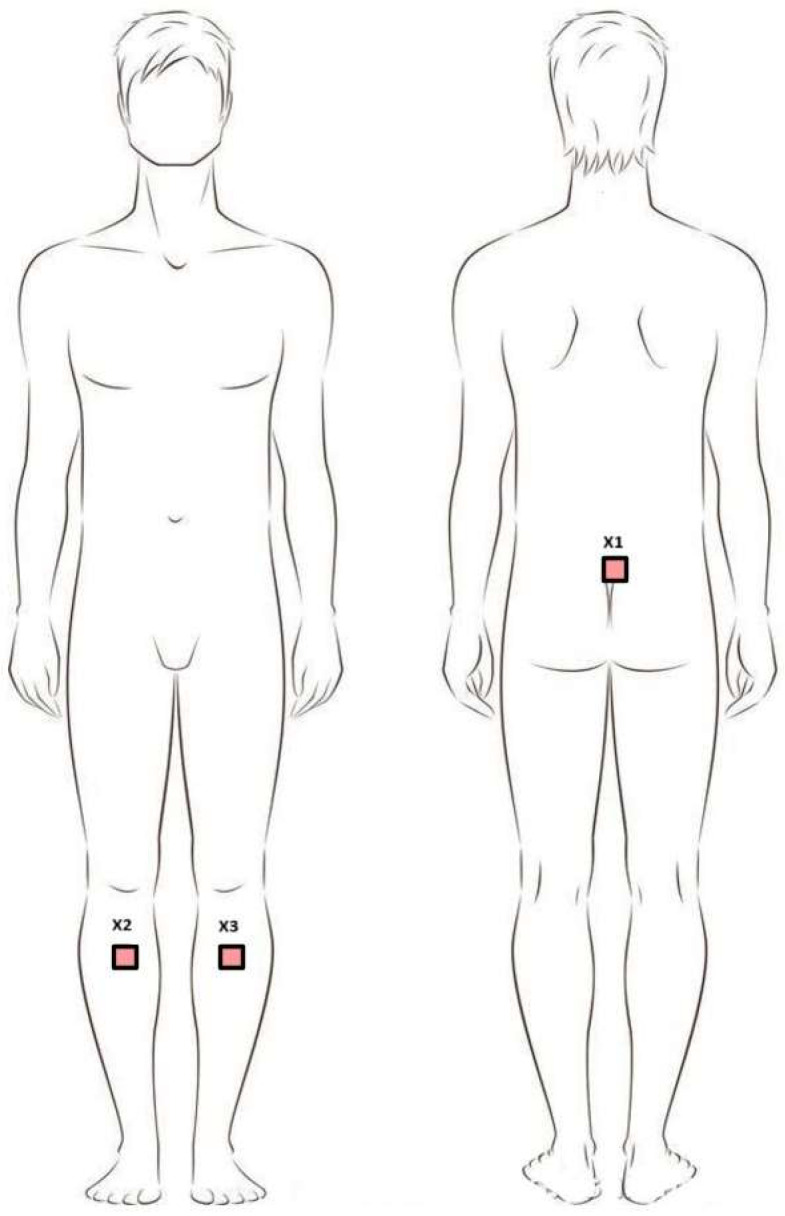
Positioning of sensors during the study. **Legend:** X1—sacral sensor; X2—sensor on the right shin; X3—sensor on the left shin.

The Movement Kinematics Analysis System (MoKa, Tychy, Poland) is a set of wearable IMU sensors, connected via Bluetooth to a tablet running dedicated software. The number and configuration of the sensors depended on the measurement purpose. Before the test was performed, IMU sensors were placed on the participants’ bodies. The device was mounted using special, dedicated, skin-safe tapes. Three sensors were used for this study. Two recorded limb movements: the lower limbs (L and R), located on the lower legs at the level of the tibial tuberosity. One sensor recorded pelvic movements: placed on the skin at the level of the sacrum with the upper edge of the sensor at the level of the lumbosacral junction (Figure 1). In our system, we used Movella DOT (previously Xsens DOT) inertial sensors, with each device integrating a 3-axis accelerometer (±16 g), a 3-axis gyroscope (± 2000°/s), and a 3-axis magnetometer (±8 Gauss). The sensor featured 64 MB of internal memory and performed internal sampling at up to 800 Hz; it outputs processed data at rates of up to 120 Hz (in recording mode) or 60 Hz in real-time streaming mode (via BLE interface). This setup allowed for precise 3D measurement of linear acceleration, angular velocity, and magnetic field, which—through a signal pipeline utilizing a strap-down integration (SDI) algorithm and calibration/filtering steps—is fused into an orientation-output (quaternions or Euler angles)-per-sensor module. The system’s strap-down integration (SDI) algorithm, implemented by the IMU sensor’s internal processor, was based on a classic inertial navigation approach, where angular velocity signals from the gyroscopes were numerically integrated to estimate the sensor’s orientation, while data from the accelerometers and magnetometers were used for long-term drift correction. Calibration procedures specific to inertial systems were also performed at this stage, including sensor bias compensation, scale factor correction, and mutual alignment of the measurement axes within the sensor system. In environments with magnetic field disturbances, magnetic field mapping was additionally required, performed using a software tool provided by the sensor manufacturer. The inertial data were filtered to reduce high-frequency noise and motion artifacts, using filters adapted to the frequency response of human gait. Data fusion was performed using an orientation estimation algorithm, which conceptually corresponded to a complementary filter or extended Kalman filter.

### 2.3. Experimental Protocol

All study participants (CG and RG) completed the 6MWT. This was a commonly used test to assess participants’ exercise (locomotion) abilities. When interpreting test results, it was important to consider participants’ gender, age (which negatively impacts results), morphological parameters, physical activity level, health status, and, according to some authors, even cultural differences, which justified research in this area [36,37]. Before the test, participants refrained from strenuous physical activity for 2 h and rested in a seated position for 10 min immediately before the test. The 6MWT was conducted on a 32-m stretch of level, flat surface.

### 2.4. Processing Date

Calibration aimed to establish zero values in further gait analysis (except for pelvic tilt), which allowed for data normalization in relation to the individual method of attaching sensors to the patient’s body. Data from IMU sensors were first segmented into time intervals and automatically classified into static (standing/sitting) and dynamic (walking, running) activities. Activities that could not be clearly assigned to any category were marked as ‘unidentified’ and excluded from further locomotion analysis. Measurements were recorded in three planes: sagittal, frontal, and transverse. Negative values recorded by the system refer to rotational movements performed to the left relative to the frontal (roll angle) and transverse (yaw angle) planes to produce right–left movements. The gait analysis algorithm included the automatic identification of successive gait cycles, determination of the limb initiating a given cycle, and assignment of corresponding pairs of right and left limb cycles or their rejection, if they do not meet the signal quality criteria. The leg initiating the cycle was determined based on characteristic changes in pelvic rotation around the vertical axis and on the angles of lower leg inclination. The angular waveforms were then normalized to the relative cycle duration (0–100%), and the average motion profiles and standard deviations were calculated based on them. Gait parameters were recorded throughout the entire duration of the test. The first straight gait segments in 1, 3, and 6 min were used for analysis. The test began with a 5-s calibration performed in the habitual position to zero the sensors, ensuring a starting position of 0°.

### 2.5. Statistical Analysis

The reliability of measurements taken at 1, 3, and 6 min was examined. Means and Standard Deviations (SD) were calculated, and the internal consistency was measured using Cronbach’s Alpha (AC) and Intraclass Correlation Coefficient (ICC). AC allowed for the calculation of the internal consistency of the test. It is the quotient of the variance of a given scale item and the total variance for the entire scale, calculated according to the formula:

$$\alpha =\frac{k}{k-1}(1-\frac{\sum_{i=1}^{k}s_{1}^{2}}{s_{c}^{2}})$$

Abbreviation: α—Alfa Cronbacha; *k*—number of test items; $s_{1}^{2}$—variance of test items; $s_{c}^{2}$—total results.

ICC is a measure of the agreement between two or more measurements. ICC is calculated according to the following steps:

1. A single-factor analysis was performed for repeated measurements.
2. Between-subject and within-subject variances were calculated.
3. Next, the ICC was calculated using the formula:

$$ICC=\frac{{MS}_{between}-{MS}_{within}}{{MS}_{between}+\left(k-1\right){MS}_{within}}$$

Abbreviation: *MS between*—average square between objects; *MS within*—average square within objects; *k*—number of measurements.

Comparisons between subsequent measurements were made using Repeated Measures ANOVA. Between-group comparisons were performed using Mann–Whitney U, and relationships between variables were determined using Spearman’s R correlations. The adopted level of significance was *p* < 0.05. Effect sizes for between-group comparisons were also calculated, using Cohen’s *d*. Statistical analyses were performed using Statistica v13 software.

## 3. Results

In the RG, the side of dysfunction had no effect on the 6MWT parameters examined. Comparison of pain intensity before and after the 6MWT revealed differences: *p* < 0.001. The RG–CG comparison revealed differences in distance and step count. There were no differences in average step length (Table 1).

In the RG, correlations between pain intensity according to the VAS and test results were examined. Pain intensity negatively correlated with distance (R = −0.44) and step count (R = −0.44), but did not affect the average step length (R = 0.05). The comparison of pain in the RG by side revealed no differences (*p* > 0.05). The overall consistency assessment (AC) demonstrated good internal consistency for three measurements and excellent for the remaining measurements. The qualitative ICC assessment indicated moderate reliability for three measurements, good for one, and excellent for the remaining measurements [38] (Table 2).

The variability of the studied parameters in individual measurements, from the 1st, 3rd, and 6th minute, was analyzed (Table 3). The changes demonstrated within the RG were observed in individuals with left knee dysfunction.

**Figure 2:**
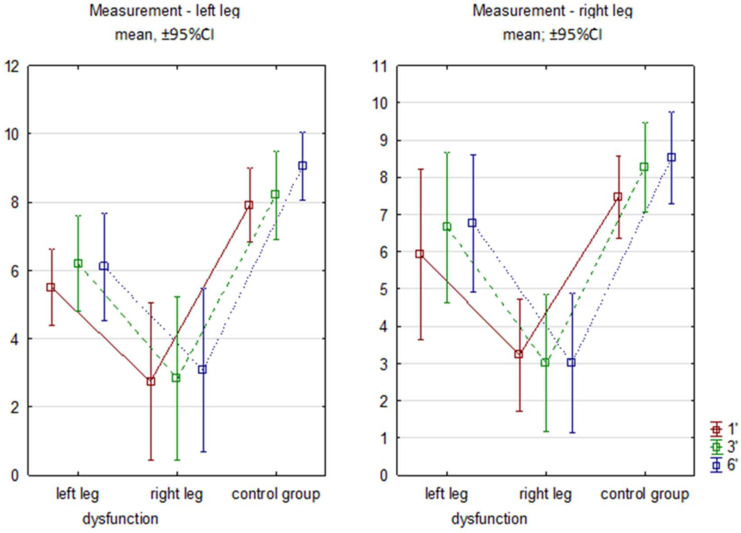
Sagittal flexion angles in subsequent minutes of 6MWT. Legend: RG—research group right/left limb; CG—control group. 1′, 3′, 6′ measurements from successive minutes of the test.

The intergroup comparison showed differences between RG, regardless of leg dysfunction, and CG at each minute and for both limbs in the sagittal plane (Figure 2).

## 4. Discussion

The epidemiology of OA and the number of arthroplasty procedures performed indicate the need for relatively inexpensive and accurate diagnostics. Considering functional parameters, the use of IMU-based systems seems to be an interesting proposition. The results presented here regarding measurement reliability seem to confirm good prospects for their widespread use. Although the AC for all measurements was good or excellent, this coefficient does not take into account all the assumptions necessary for repeated biomechanical measurements. The qualitative assessment of the more reliable ICCs was slightly lower, but the results appeared satisfactory. The moderate reliability in the transverse plane was likely due to heterogeneity among the subjects. This presented a challenge for further research in this area.

The Osteoarthritis Research Society International recommends that individuals with a confirmed diagnosis of knee OA be monitored for improvement in three specific and important activities of daily living: (1) rising from a chair, (2) climbing stairs, and (3) walking. These movements are clinically important because they are associated with pain, stiffness, and limited ability to participate in social activities [17]. This justifies studies using the 6 MWT in individuals with OA and comparing their results with those of a healthy population. In a study by Camarri et al., conducted on a population of 70 healthy Caucasian individuals aged 64.5 ± 5.2 years, the results ranged from 484 to 820 m in men and were 59 ± 13 m higher than in women [39]. In turn, Bohannon, in his meta-analysis, presented reference values for women aged 60–69 years (460–549 m) and for women aged 70–79 years (442–538 m) [40]. Despite the optimal conditions used in our study (straight line length—32 m), the study results were lower than those that the authors presented [41]. In the RG, the explanation was KOA, and, in particular, the severity of pain. According to Bejek et al., the highest walking speed of patients with severe osteoarthritis—without pain or loss of coordination—was 2 km/h [42]. The speed of the women studied in the present study exceeded 3 km/h, which explained the severity of pain after performing the 6 MWT. The results of the presented study also confirmed the observations regarding the effect of excess body weight on KOA, and consequently on the 6 MWT result. They were consistent with the results of the study by Millar et al. [43]. In the CG, the result was most likely influenced by the progressive process of sedentary behavior in many societies, including Poland.

No differences were noted between the RG and CG regarding step length; the difference in distance was influenced by the number of steps, which clearly indicated a slower gait in individuals with KOA, which was a manifestation of an adaptive mechanism. In this context, it is worth mentioning the concept of “energy capacity” as a factor limiting the activity of older people [44]. The decline in total energy capacity with age—combined with an inefficient gait pattern and pain, which can also cause an increase in energy expenditure—consequently affects the limitation of health-beneficial activity levels [45,46]. Therefore, the limited mobility associated with OA was a poor prognostic factor for overall health [47]. This justified both preventive measures, surgical interventions if necessary, and research into biomechanical gait analysis.

Due to OA, individuals with OA adopted different gait strategies. No differences were found in the studied parameters based on the side qualifying for surgery in the RG. We believe this was a manifestation of compensation aimed at achieving gait symmetry, which determined a safe gait and optimal energy expenditure [23]. Comparing subsequent minutes of walking in the RG, changes were noted only in the group with left knee dysfunction. In this case, a correction occurred in the medial direction of the right limb. We suppose that this mechanism was intended to reduce any pain experienced by relieving the affected limb. In the intergroup comparisons between the RG and CG, differences in the sagittal plane were particularly noticeable between both limbs. Higher CG values indicated less stiffness and greater gait dynamics. This might also explain the observed changes in individual minutes of walking in this group. The flattening and slowing of gait in the RG might also be explained by a mechanism aimed at maintaining gait rhythm. This finding was analogous to our previous studies on women with OA of the hip joints [37].

Kushioka et al. conducted the 6MWT with IMU sensors in a group of individuals with KOA and lumbar spinal stenosis (LSS). They performed a minute-by-minute analysis of the 6MWT in each group. The authors placed the sensors exclusively on the subjects’ feet. They observed that, in the third minute of the 6MWT, the coefficient of variation in step length in the LSS group was significantly higher than that in the KOA group. Furthermore, the LSS group showed a trend toward greater step length variability in the fourth minute of the 6MWT. This increased step length variability in the LSS group indicated a deterioration in gait rhythmicity. Gait variability, or fluctuations in step length during walking, is a potential biomarker of gait disorders and loss of gait rhythmicity. An increase in variability indicated a deterioration in gait consistency. Studies have shown that maintaining gait consistency is a complex process dependent on various neurological structures, from the cerebral cortex to peripheral nerves and muscles. Therefore, damage to any neuromuscular structure responsible for locomotor control might result in loss of gait rhythm [48,49]. These observations seem important from the perspective of patients’ functioning after joint replacement and physiotherapy. Compensations and movement habits can influence gait patterns even after the removal of structural abnormalities (joint replacement) and are independent of the patient’s perceptions, which is why objectivity of the research is such a crucial aspect [21].

In our opinion, the results of the present study using the MoKa system, which met the criteria of simplicity, minimal cost, and reliability, can provide valuable information on the gait characteristics of people with KOA, which is consistent with the increasingly widespread use of wearable sensors in clinical applications [17].

### Limitations

The limitations of the study were typical for cross-sectional studies and suggested the need for further research aimed at long-term observations, including patients after surgery, concerning the relationship between gait parameters and pain reduction in patients after surgery or, more generally, lifestyle and quality of life. The number of subjects should also be larger, as indicated by Cohen’s d coefficient. The development of indicators that measure the deviation of a subject’s gait pattern from the typical pattern is worth considering in order to assess the progress of therapy. Another limitation was the fact that in our study, a single person was tasked with attaching the sensors to the subjects’ bodies. Repeated measurements by different people, while maintaining a precise research procedure, could confirm the reliability of the results obtained.

## 5. Conclusions

According to the reliability indicators presented, the measurement of biomechanical gait parameters using a system equipped with an IMU meets the measurement reliability criteria. The presented results of the gait parameter in the sagittal plane indicate that its use opens the door to further research and application in physiotherapy planning for individuals with KOA, whose aim would be to achieve the best possible gait parameters, while maintaining symmetry and minimal energy expenditure. The gait of women with OA undergoing joint replacement surgery was flattened and slowed, which might provide guidance for the use of appropriate post-operative therapies to achieve appropriate gait biomechanics. Due to the advantages presented, the use of the MoKA system equipped with an IMU seems to be the optimal method for measuring gait parameters in people with KOA.

## Figures and Tables

**Table 1:** Results of the 6MWT in the research and control groups.

Parameter	Research Group				Control Group		Research–Control: *p*
	Dysfunctional Leg	Mean (±95% CI)	Median	Person with Dysfunction Left–Right: *p*	Mean (±95% CI)	Median	
6MWT average step length [m]	left	303 (213–393)	307		360 (330–391)	371	**
	right	291 (259–323)	294				
6MWT number of steps [n]	left	499 (370–627)	528		595 (554–636)	618	*
	right	524 (467–581)	531				
6MWT average step length [m]	left	0.6 (0.4–0.7)	0.6		0.6 (0.5–0.6)	0.6	
	right	0.6 (0.5–0.6)	0.5				

* *p* < 0.05; ** *p* < 0.01.

**Table 2:** Reliability of the measurements performed.

Plane	Part of the Body	Mean (SD)	AC	ICC
frontal	left leg	−0.12 (3.33)	0.95	0.87
	right leg	0.73 (4.64)	0.96	0.90
	pelvis	0.02 (1.18)	0.88	0.71
sagittal	left leg	7.56 (3,46)	0.97	0.92
	right leg	7.26 (3.38)	0.97	0.90
	pelvis	21.51 (7.94)	0.99	0.98
transverse	left leg	−1.62 (9.56)	0.80	0.57
	right leg	1.59 (7.16)	0.81	0.59
	pelvis	0.01 (0.21)	0.99	0.99

**Table 3:** Results of the tested gait parameters in individual measurements.

Measurement		Time	Research Group (RG)				Control Group (CG)		RG-CG:	
Part of the Body	Plane		Dysfunction							
			Left Leg		Right Leg				*p* ^[2]^	Cohen’s *d*
			Mean (±95% CI)	*p* ^[1]^	Mean (±95% CI)	*p* ^[1]^	Mean (±95% CI)	*p* ^[1]^		
left leg	frontal	1′	0.1 (−1.0–1.1)		−0.1 (−2.0–1.8)		−0.4 (−1.1–0.3)	***		−0.1
		3′	1.1 (−0.3–2.4)		−1.3 (−4.8–2.2)		0.1 (−0.8–0.9)			0.3
		6′	1.3 (−0.4–2.9)		−1.2 (−4.7–2.4)		0.3 (−0.6–1.2)			−0.3
right leg		1′	1.7 (−0.1–3.3)	***	1.6 (0.0–3.3)		1.2 (0.4–2.0)			0.0
		3′	0.7 (−1.4–2.8)		1.2 (−1.6–3.9)		0.7 (0.5–1.9)			0.2
		6′	0.5 (−1.6–2.6)		−2.1 (−9.5–5.3)		0.6 (−0.6–1.9)			0.2
left leg	sagittal	1′	5.5 (4.4–6.6)		2.7 (0.5–5.0)		8.2 (7.4–9.0)	***	****	0.4
		3′	6.2 (4.8–6.6)		2.8 (0.4–5.2)		8.6 (7.6–9.7)		***	0.3
		6′	6.1 (4.5–7.7)		3.1 (0.7–5.5)		9.0 (8.0–10.1)		****	0.9
right leg		1′	5.9 (3.6–8.2)		3.7 (2.6–4.8)		7.8 (6.9–8.7)	*	***	0.2
		3′	6.7 (4.6–8.7)		3.0 (1.2–4.9)		8.2 (6.9–9.4)		***	0.2
		6′	6.8 (4.9–8.6)		3.9 (2.8–5.1)		8.9 (8.0–9.8)		****	0.3
left leg	transverse	1′	−1.2 (−3.3–0.9)		0.7 (−2.4–3.8)		2.3 (1.1–3.5)		*	0.9
		3′	−5.7 (−14.8–3.4)		−1.4 (−6.5–3.7)		1.4 (−0.6–3.4)			0.5
		6′	−3.9 (−19.4–11.7)		−0.0 (−4.9–4.8)		1.5 (−2.2–5.2)			0.3
right leg		1′	0.5 (−3.1–4.0)		−1.6 (−5.0–1.9)		1.3 (0.2–2.5)	**		−0.1
		3′	−4.2 (−15.4–7.1)		−1.1 (−7.8–5.6)		3.7 (1.0–6.4)		*	0.5
		6′	−9.2 (−25.6–7.3)		0.3 (−11.5–12.2)		6.1 (2.3–9.9)		*	1.0
pelvis	frontal	1′	0.5 (−0.6–1.6)		0.2 (−0.9–1.3)		−0.1 (−0.5–0.3)			−0.6
		3′	0.4 (−0.5–1.4)		−0.6 (−2.0–0.7)		−0.1 (−0.6–0.5)			−0.4
		6′	0.9 (0.1–1.8)		−2.2 (−4.9–0.6)		−0.2 (−0.7–0.3)			−0.6
	sagittal	1′	20.4 (15.7–25.1)		20.1 (14.5–25.6)		22.2 (20.2–24.1)			0.1
		3′	22.0 (18.5–25.5)		20.3 (15.0–25.6)		22.1 (20.1–24.0)			−0.1
		6′	22.2 (18.5–25.8)		20.7 (15.5–26.0)		22.5 (20.5–24.4)			0.0
	tranverse	1′	−0.1 (−0.2–−0.1)		−0.1 (−0.3–0.2)		0.1 (−0.0–0.1)			0.7
		3′	−0.0 (−0.1–0.1)		−0.1 (−0.5–0.4)		0.0 (−0.1–0.1)			0.1
		6′	−0.0 (−0.2–0.1)		0.3 (0.0–0.6)		0.0 (−0.1–0.1)			0.2

* *p* < 0.05; ** *p* < 0.01; *** *p* < 0.001; **** *p* < 0.0001; ^[1]^ Repeated Measures ANOVA: comparison 1′-3′-6′; ^[2]^ U Mann–Whitney test: comparison RG-CG.

## Data Availability

The raw data supporting the conclusions of this article will be made available by the authors on request.
